# Divergent Myelination or Divergent Trajectories? Insights from MPF Mapping in Bipolar Disorder and Recurrent Depressive Disorder

**DOI:** 10.3390/bioengineering13020243

**Published:** 2026-02-19

**Authors:** Remigiusz Recław, Anna Grzywacz

**Affiliations:** 1 Independent Laboratory of Genetics and Behavioral Epigenetics, Pomeranian Medical University in Szczecin, Powstańców Wielkopolskich 72 Street, 70-111 Szczecin, Poland; grzywacz.anna.m@gmail.com; 2 Department of Medical Sciences and Public Health, Gdansk University of Physical Education and Sport, Kazimierza Górskiego 1 Street, 80-336 Gdansk, Poland

**Keywords:** macromolecular proton fraction, white matter, bipolar disorder, recurrent depressive disorder, myelination, quantitative MRI

## Abstract

Quantitative magnetic resonance imaging has increasingly highlighted white matter abnormalities as a key component of affective disorders. Fast macromolecular proton fraction (MPF) mapping, a myelin-sensitive technique, recently revealed divergent patterns of white matter myelination in bipolar disorder (BD) and recurrent depressive disorder (RDD), with reduced MPF in RDD but elevated MPF in BD. These findings challenge uniform hypomyelination models of mood disorders. In this Communication, we propose a trajectory-oriented reinterpretation of these results, suggesting that MPF differences may reflect distinct neurodevelopmental and lifespan-related myelination trajectories rather than a simple marker of tissue damage. Elevated MPF in BD—observed particularly in relatively young patients—may indicate accelerated or dysregulated white matter maturation or activity-dependent myelin plasticity, whereas reduced MPF in RDD may reflect impaired maintenance of myelin integrity. We emphasize that MPF should not be interpreted as a unidirectional index of pathology and argue that it may serve as a phenotype-differentiating biomarker between BD and RDD, warranting further longitudinal and multimodal studies.

Recent advances in quantitative MRI have renewed interest in white matter abnormalities as a potential neurobiological substrate of affective disorders, particularly in light of growing evidence that myelination is a dynamic and experience-dependent process rather than a static structural feature of the brain [1]. In this context, the study by Gusakova et al. [2] provides a valuable and methodologically robust contribution by applying a fast acquisition protocol for macromolecular proton fraction (MPF) mapping to directly compare white matter myelination in bipolar disorder and recurrent depressive disorder. By demonstrating reduced MPF in recurrent depressive disorder alongside markedly elevated MPF in bipolar disorder, the authors challenge simplified models of uniform hypomyelination across affective conditions. These findings invite further discussion regarding the biological interpretation of MPF differences and their implications for understanding divergent pathophysiological processes underlying both affective conditions, particularly within a developmental framework [3]. Unlike the original empirical report, the present communication aims to provide a trajectory-oriented and developmental reinterpretation of these findings, positioning MPF differences within a broader lifespan and neurodevelopmental framework.

The most striking finding is the opposite direction of MPF changes: hypomyelination in recurrent depressive disorder versus elevated MPF in bipolar disorder (both relative to controls and to each other). This pattern stands in contrast to the prevailing view that white matter pathology in affective disorders is predominantly characterized by global myelin loss, a perspective largely informed by diffusion-based imaging studies and meta-analytic evidence in major depressive disorder [4]. Importantly, the elevation in MPF in bipolar disorder should not be regarded as a trivial or incidental finding [5], as it suggests that white matter alterations in mood disorders may follow qualitatively different biological trajectories rather than reflecting a shared deficit. Such divergence raises the question of whether MPF differences primarily index pathological damage or instead capture dynamic, state- or development-dependent processes within the myelination continuum.

One plausible framework for interpreting these findings is a neurodevelopmental and trajectory-based perspective on white matter myelination in affective disorders. Rather than reflecting a simple presence or absence of myelin pathology, MPF differences may capture alterations in the timing, rate, or regulation of myelination across the lifespan—processes known to be particularly sensitive during adolescence and early adulthood [3,6]. In this context, the elevated MPF in bipolar disorder (observed in a relatively young patient group, median age ≈24 years) could reflect accelerated or dysregulated white matter maturation rather than a protective or “hypermyelinated” state per se, consistent with reports of altered age-related white matter trajectories in bipolar disorder [5].

A related mechanistic explanation for this elevated MPF may involve activity-dependent myelination driven by repeated engagement of limbic–prefrontal circuits, particularly during depressive phases of bipolar disorder, which have been associated with heightened limbic activity and reduced prefrontal regulation. While this hypothesis is biologically plausible given known mechanisms of experience- and activity-dependent plasticity in oligodendrocytes and myelin formation [1], it remains speculative and would require longitudinal studies combining MPF mapping with functional connectivity measures (e.g., resting-state fMRI) to test directly. Conversely, the reduced MPF in recurrent depressive disorder may indicate a distinct trajectory characterized by impaired maintenance or progressive disruption of myelin integrity, in line with quantitative MRI evidence of reduced myelination in depressive disorders [7].

From a biomarker perspective, these findings underscore the need for caution in interpreting MPF as a unidirectional indicator of white matter pathology in affective disorders. While reduced MPF is often intuitively equated with demyelination or tissue damage, elevated MPF values—such as those observed in bipolar disorder—may reflect compensatory, state-dependent, or developmentally modulated processes rather than improved white matter integrity. This distinction is particularly relevant given the biophysical specificity of MPF as a myelin-sensitive metric and its fundamental differences from diffusion-based indices [8,9,10]; in this context, DTI and MPF probe different components of white matter microstructure, which may partly explain divergent directions of effects across imaging modalities. In the two-pool magnetization transfer model, MPF quantifies the relative fraction of protons bound to macromolecules, primarily myelin-associated lipids and proteins, that exchange magnetization with free water protons. These non-unidirectional neuroimaging findings are consistent with broader evidence of atypical neurodevelopmental trajectories and microstructural organization in bipolar disorder, including meta-analytic reports of minor physical anomalies and increased cortical gray–white matter contrast across independent MRI studies [11,12]. Thus, MPF mapping holds greater promise as a phenotype-differentiating biomarker (bipolar disorder vs. recurrent depressive disorder) than as a nonspecific index of disease severity, with clear potential for aiding early diagnostic differentiation. Notably, the reported correlations between MPF values and symptom severity explained only a limited proportion of variance, consistent with correlation coefficients corresponding to approximately 10–20% of explained variance, indicating that additional developmental and environmental factors likely contribute to MPF variability. It is worth noting that MPF differences may be partly modulated by the current illness phase and pharmacotherapy (especially mood stabilizers in BD), thereby reinforcing the need for longitudinal studies accounting for these factors [13,14,15,16].

In summary, the work by Gusakova et al. [2] provides an important empirical foundation for rethinking white matter alterations in affective disorders beyond simplified deficit-based models. Their findings highlight the potential value of MPF mapping in capturing biologically meaningful heterogeneity between bipolar disorder and recurrent depressive disorder, while also emphasizing the need for longitudinal and multimodal approaches to fully elucidate the underlying mechanisms. Importantly, the original study was based on relatively modest group sizes and a cross-sectional design, underscoring the need for large-scale, longitudinal investigations to confirm the robustness and reproducibility of the reported effects. Future studies integrating MPF with diffusion-based metrics, clinical staging, and developmental trajectories may help clarify whether observed differences reflect transient states, compensatory adaptations, or enduring neurobiological signatures across the lifespan [5,6]. Such efforts are crucial to advancing a truly trajectory-oriented, lifespan-informed understanding of white matter heterogeneity in mood disorders.

## Data Availability

No new data were created or analyzed in this study.

## References

[1] Fields R.D. (2008). White Matter in Learning, Cognition and Psychiatric Disorders. Trends Neurosci..

[2] Gusakova S., Smirnova L., Borodin O., Epimakhova E., Seregin A., Yarnykh V. (2026). Macromolecular Proton Fraction Reveals Divergent White Matter Myelination in Bipolar Disorder and Unipolar Recurrent Depression. Bioengineering.

[3] Paus T., Keshavan M., Giedd J.N. (2008). Why Do Many Psychiatric Disorders Emerge during Adolescence?. Nat. Rev. Neurosci..

[4] Liao Y., Huang X., Wu Q., Yang C., Kuang W., Du M., Lui S., Yue Q., Chan R.C., Kemp G.J. (2013). Is Depression a Disconnection Syndrome? Meta-Analysis of Diffusion Tensor Imaging Studies in Patients with MDD. J. Psychiatry Neurosci..

[5] Tønnesen S., Kaufmann T., Doan N.T., Alnæs D., Córdova-Palomera A., Meer D.V., Rokicki J., Moberget T., Gurholt T.P., Haukvik U.K. (2018). White Matter Aberrations and Age-Related Trajectories in Patients with Schizophrenia and Bipolar Disorder Revealed by Diffusion Tensor Imaging. Sci. Rep..

[6] Kochunov P., Glahn D.C., Lancaster J., Thompson P.M., Kochunov V., Rogers B., Fox P., Blangero J., Williamson D.E. (2011). Fractional Anisotropy of Cerebral White Matter and Thickness of Cortical Gray Matter across the Lifespan. NeuroImage.

[7] Sacchet M.D., Gotlib I.H. (2017). Myelination of the Brain in Major Depressive Disorder: An in Vivo Quantitative Magnetic Resonance Imaging Study. Sci. Rep..

[8] Yarnykh V.L. (2012). Fast Macromolecular Proton Fraction Mapping from a Single Off-Resonance Magnetization Transfer Measurement. Magn. Reson. Med..

[9] Kisel A.A., Naumova A.V., Yarnykh V.L. (2022). Macromolecular Proton Fraction as a Myelin Biomarker: Principles, Validation, and Applications. Front. Neurosci..

[10] Underhill H.R., Yuan C., Yarnykh V.L. (2009). Direct Quantitative Comparison between Cross-Relaxation Imaging and Diffusion Tensor Imaging of the Human Brain at 3.0 T. NeuroImage.

[11] Varga E., Hajnal A., Soós A., Hegyi P., Kovács D., Farkas N., Szebényi J., Mikó A., Tényi T., Herold R. (2021). Minor Physical Anomalies in Bipolar Disorder—A Meta-Analysis. Front. Psychiatry.

[12] Jørgensen K.N., Nerland S., Norbom L.B., Melle I., Agartz I., Andreassen O.A., Westlye L.T., Dale A.M. (2016). Increased MRI-Based Cortical Grey/White-Matter Contrast in Sensory and Motor Regions in Schizophrenia and Bipolar Disorder. Psychol. Med..

[13] Nortje G., Stein D.J., Radua J., Mataix-Cols D., Horn N. (2013). Systematic Review and Voxel-Based Meta-Analysis of Diffusion Tensor Imaging Studies in Bipolar Disorder. J. Affect. Disord..

[14] Xu M., Zhang W., Hochwalt P., Yang C., Liu N., Qu J., Sun H., DelBello M.P., Lui S., Nery F.G. (2022). Structural Connectivity Associated with Familial Risk for Mental Illness: A Meta-Analysis of Diffusion Tensor Imaging Studies in Relatives of Patients with Severe Mental Disorders. Hum. Brain Mapp..

[15] Favre P., Pauling M., Stout J., Fukunaga M., Alnæs D., Baur-Streubel R., Biedermann N., Boraxbekk D.M., Dima D., Doan N.T. (2019). Widespread White Matter Microstructural Abnormalities in Bipolar Disorder: Evidence from Mega- and Meta-Analyses across 3033 Individuals. Neuropsychopharmacology.

[16] Chen G., Hu X., Li L., Wang S., Chen C., Guo J., Wang C., Yang X., Lui S., Gong Q. (2016). Disorganization of White Matter Architecture in Major Depressive Disorder: A Meta-Analysis of Diffusion Tensor Imaging with Tract-Based Spatial Statistics. Sci. Rep..

